# Characterization and reutilization potential of lipids in sludges from wastewater treatment processes

**DOI:** 10.1038/s41598-020-69855-6

**Published:** 2020-08-03

**Authors:** Shuai Liu, Tao Luo, Guo-hua Liu, Xianglong Xu, Yuting Shao, Lu Qi, Hongchen Wang

**Affiliations:** Low-Carbon Water Environment Technology Research Center, School of Environment and Natural Resources, Renmin University of China, Beijing, 100872 China

**Keywords:** Environmental sciences, Environmental chemistry

## Abstract

Lipids in sewage sludge are considered to be high-class raw materials for biodiesel or other valuable products. We profiled the characteristics and assessed the reutilization potential of lipids from different sludge sources, including sludge from the primary sedimentation tank (PST sludge) and sludge from the secondary sedimentation tank in a conventional activated sludge system (CAS sludge), as well as sludge from ultrashort-sludge retention time (SRT) activated sludge systems with different SRTs (USAS sludge, with SRTs of 0.5, 1.0, 2.0, 3.0 and 4.0 d). The results showed that the lipids in the sludges were mainly composed of cellular lipids, free fatty acids (FFAs), wax and gum. The highest lipid content was found in the PST sludge (156.8 ± 11.9 mg/g, dry basis), followed by the USAS sludges (67.9 ± 11.0–132.2 ± 11.8 mg/g) and the CAS sludge (46.0 ± 16.5 mg/g). Lipid species such as Cer, So, PE, PC, and TG were abundant, comprising over 80% of the cellular lipids in the sludges. With higher lipid contents, the PST sludge and USAS sludge (0.5 d SRT) were suggested to have a higher reutilization potential for use in producing biodiesel. In addition, the CAS sludge was promising for resource reutilization and energy recovery due to the large amount of excess sludge.

## Introduction

By the end of June 2017, over 3,700 wastewater treatment plants (WWTPs)^1^ had been built and operated in China, and a large amount of sludge was produced each year. Based on a report from the China Statistical Yearbook on Environment (2018), the yield of municipal sludge with 80% moisture content increased from 33.18 billion tons in 2008 to 49.24 billion tons in 2017^1^. Thus, sludge treatment has become an urgent issue in China, and the main treatment method is landfilling. However, sludges from the primary and secondary sedimentation tanks in WWTPs contain a large amount of organic matter^2^, which has the potential for resource and energy recovery. In particular, the concentration of lipids in sludge has been reported to be high, accounting for approximately 20% of the organic matter^3^, and thus, lipids are considered to be a potential feedstock for use in diesel production^4–8^. The lipids in municipal sewage sludge include cellular lipids, free fatty acids, wax and gum, the contents and diesel production potential of which are affected by the sludge source.

It has been reported that the biodiesel yield of sludge from the primary sedimentation tank (PST) is much higher than that of sludge from the secondary sedimentation tank in the conventional activated sludge (CAS) process due to substrate utilization by the metabolism of microorganisms in the bioreactor^9^. One possible approach to achieve resource recovery from sludge in WWTPs is to prevent the substrates from being utilized by microorganisms by reducing the sludge retention time (SRT) in the CAS process. Recently, an ultrashort-SRT activated sludge (USAS) process was developed for organic recovery; this process originates from the A stage of the adsorption-biodegradation (AB) process, where the A stage is operated with a high organic load and enables the high recovery of organic matter by biosorption rather than degradation^10,11^. Therefore, sludge from the USAS process is also considered to be a potential raw material for energy recovery. Studies by Trzcinski et al.^12^ and Meerburg et al.^13^ suggested that sludge obtained from the A stage achieved a higher methane yield than that from B stage by 1.4–1.9 times during the anaerobic digestion process. However, few studies have focused on the analysis of lipids from USAS sludges.

To better understand the potential for the reuse of lipids from different kinds of sludge, detailed analysis of the characteristics of the lipids is necessary. In this study, sludges produced from the PST and SST in a CAS process, as well as from USAS processes with different SRTs, were used to perform lipidomic analysis using Soxhlet extraction and ultrahigh-performance liquid chromatography-tandem mass spectrometry (UPLC-MS/MS).

## Materials and methods

### Sludge

The PST sludges were collected from a tank containing sewage from the campus of Renmin University of China (Beijing) after settling for 1.5 h. The CAS sludges were collected from the secondary sedimentation tank of a lab-scale anaerobic-anoxic–oxic (A^2^/O) activated sludge system (12 d SRT). The USAS sludges were collected from the secondary sedimentation tanks of five ultrashort-SRT activated sludge systems where the SRT was set to 0.5, 1.0, 2.0, 3.0, and 4.0 d. All the reactors were inoculated with sludge from the Xiaohongmen wastewater treatment plant (Beijing), and the two types of activated sludge systems were both fed with sewage that had settled in the PST for 1.5 h.

## Experimental methods

### Pretreatment

All sludge samples (total solid concentration (TS): 1.0% ± 0.5%) were centrifuged (XiangYi L-530, XiangYi, Changsha) at 3,000 rpm for 10 min for preliminary dehydration to obtain dewatered sludge (TS: 15.0% ± 0.5%). Further freeze-drying of the dehydrated sludge included the following steps: the dehydrated sludges were frozen in a − 20 °C refrigerator for 48 h, and then the frozen dehydrated sludges were placed in an automatic vacuum freeze dryer (FD-1A-50, Boyikang, Beijing) and freeze-dried for 48 h. The sludge samples obtained after freeze-drying were crushed using a mortar and pestle and then stored in a refrigerator (− 4 °C) for subsequent analysis.

### Extraction methods

Mixtures of chloroform and methanol have been widely used as lipid extractants: a monophasic solution is obtained when sludge is homogenized with a mixture of chloroform and methanol, and the homogenate can be diluted with water and/or chloroform to obtain a biphasic system; the chloroform layer, which could contain lipids, is separated to extract purified lipids^14,15^. Hence, by adding different proportions of chloroform–methanol-water before and after dilution, cellular lipids and free fatty acids can be extracted.

#### Extraction of cellular lipids

The modified method used to extract cellular lipids was adapted from the method of Folch et al.^14^. Briefly, a 1 g sample of dry sludge was put into a 100 mL stoppered centrifuge tube (PTFE). Then, 2 mL of citric acid buffer (0.15 M, pH = 7.3) and 7.5 mL of methanol–chloroform (1:2) were added, and the solution was mixed on a horizontal oscillator (SHA-CA, Ronghua Instruments, China) for 2 h of extraction. After that, 6 mL of a methanol-chloroform (1:2) mixture and 4.5 mL of citric acid buffer were added to the extraction solution. The mixture was mixed thoroughly on a vortex oscillator (VORTEX-5, Kylin-Bell, China) and allowed to sit undisturbed overnight for phase separation. The sample was centrifuged (SiGMA @ 3 K-15, SiGMA, PA) at 4,000 rpm for 10 min, and then the upper organic phase containing lipids was transferred to a 10 mL heat-resistant glass tube. The lipid was separated by a nitrogen stripper (NDK200-2 N, MIULAB, China) and stored in a refrigerator at − 4 °C for further analysis.

#### Extraction of free fatty acids

The modified extraction method for free fatty acids (FFAs) was mainly based on the method of Bligh and Dyer^15^. A 1 g sample of dry sludge was put into a 50 mL stoppered centrifuge tube (PTFE). Then, 1.5 mL of citric acid buffer (0.15 M, pH = 7.3), 1.9 mL of chloroform, and 3.75 mL of methanol were added, and the mixture was mixed thoroughly on a vortex oscillator. After that, the solution was oscillated at 250 rpm for 2 h in a fully automatic flip oscillator in the dark and centrifuged (SiGMA @ 3 K-15, SiGMA, PA) at 4,000 rpm for 5 min. After centrifugation, the supernatant was collected. Then, 7.6 mL of a methanol-chloroform (2:1) mixture was added to the remaining sludge residue, and the mixture was mixed thoroughly in a vortex oscillator. After that, the solution was placed in a horizontal oscillator (SHA-CA, Ronghua Instruments, China) for 2 h and then centrifuged (SiGMA @ 3 K-15, SiGMA, PA) at 4,000 rpm for 5 min. The supernatant was mixed with the previously obtained supernatant, 6 mL of a methanol-chloroform (2:1) mixture and 4.8 mL of citric acid buffer (0.15 M, pH = 7.3) were added, and then the mixture was oscillated in a vortex oscillator (VORTEX-5, Kylin-Bell, China) and incubated overnight for phase separation. The lower layer of liquid (chloroform) containing free fatty acids was transferred to a heat-resistant glass tube, separated with a nitrogen stripper (NDK200-2 N, MIULAB, China) and stored in a refrigerator at − 4 °C.

#### Extraction of wax and gum

The extraction of wax and gum was mainly based on the method adopted by Rajam and Huynh et al.^16,17^. First, the Soxhlet extraction method was used to extract lipids from the dry sludge, and the extracted lipids were dissolved in acetone and concentrated at 60 °C for 1 h. After cooling to room temperature, the wax and gum crystallized after being left in a refrigerator at 5 °C for 24 h. The solution was then centrifuged in a high-speed centrifuge (SiGMA @ 3 K-15, SiGMA, PA) at 5 °C and 8,000 rpm, and the liquid and solid components were separated by vacuum filtration. The above steps were repeated for the filtrate to ensure the complete extraction of wax and gum. Finally, the separated wax and gum were weighed, and no further analysis was performed.

### Detection and analysis methods

Ultrahigh-performance liquid chromatography with mass spectrometry (UPLC-MS/MS) was applied for the further lipidomic analysis of cellular lipids and free fatty acids. Before analysis, the extracted lipids were reconstituted with chloroform and analyzed according to the method described by Tang et al.^18^. A Q-Exactive UPLC-MS/MS (Thermo Fisher, CA) system equipped with a heated electrospray ionization probe was used. A Hypersil GOLD C18 100 × 2.1 mm × 1.9 μm column (Thermo Fisher, CA) was used to separate the lipid extracts, and samples were injected by a UPLC pump coupled to a Thermo Fisher Scientific autosampler. A flow rate of 250 μL/min was used in the analysis, with a column temperature of 45 °C and a sample temperature of 10 °C. For the binary solvent system, phase A (ACN:H_2_O = 60:40 with 10 mM ammonium acetate) and phase B (IPA:ACN = 90:10 with 10 mM ammonium acetate) were used as the mobile phase. The separations were performed for over 30 min using the program listed in Supplementary Table S3 online. The mass spectrometry conditions for cellular lipids and free fatty acids were as follows:

#### Cellular lipids

The mass spectrometry conditions were mainly based on the analytical methods of Zhu et al.^21^. Data in positive and negative ion modes were acquired through data-dependent MSMS acquisition with different mass ranges of mass/charge (m/z) values: 240–2000 and m/z 200–2000, respectively. Full-scan mode with a resolution of 70,000 was used, and fragment spectra with a resolution of 17,500 were collected. The source parameters were as follows: spray voltage: 3,000 V; capillary temperature: 320 °C; heater temperature: 300 °C; sheath gas flow rate: 35 arb; auxiliary gas flow rate: 10 arb. Lipidomic identification was performed using the analytical software Lipid Search 4.0 (Thermo Fisher, CA).

#### Free fatty acids

The mass spectrometry conditions were mainly based on the analytical methods of Zhu et al.^21^. Data in negative ion mode were acquired through data-dependent MSMS acquisition with different mass ranges of mass/charge (m/z) values: 240–2000 and m/z 200–2000. Full-scan mode with a resolution of 70,000 was used, and fragment spectra with a solution of 17,500 were collected. The source parameters were as follows: spray voltage: 3,000 V; capillary temperature: 320 °C; heater temperature: 300 °C; sheath gas flow rate: 35 arb; auxiliary gas flow rate: 10 arb. Lipidomic identification was performed using the analytical software Lipid Search 4.0 (Thermo Fisher, CA).

For the analysis of cellular lipids, some lipids, including ceramide (Cer), phosphatidylcholine (PC), cardiolipin (CL), phosphatidylethanolamine (PE), and lysophosphatidylcholine (LPC), were determined using the area external standard method, while others, such as phosphatidylglycerol (PG), phosphatidylinositol (PI), triglycerides (TG), diglycerides (DG), sphingosine (So), dimethyl phosphatidylethanolamine (dMePE), (0-acyl)-1-hydroxy fatty acid (OAHFA) and coenzyme (Co), were analyzed by a semiquantitative method based on the peak area. Different lipids respond differently in positive or negative ion mode: TG, DG, and PC have a better response in positive ion mode, while PE, PI, and CL can be detected in negative ion mode. For the analysis of the content of free fatty acids, the content was determined by an external standard method, and the standard was arachidonic acid-d8. More than one kind of different molecule was detected for each lipid species; for instance, 36 different PC molecules and 131 different TG molecules were detected. The amount of each lipid was the sum of the detected molecules, and the relative content was determined with respect to the dry sludge used for the analysis.

## Results and discussion

### Total concentration of lipids in the PST, CAS, and USAS sludges

**Figure 1:**
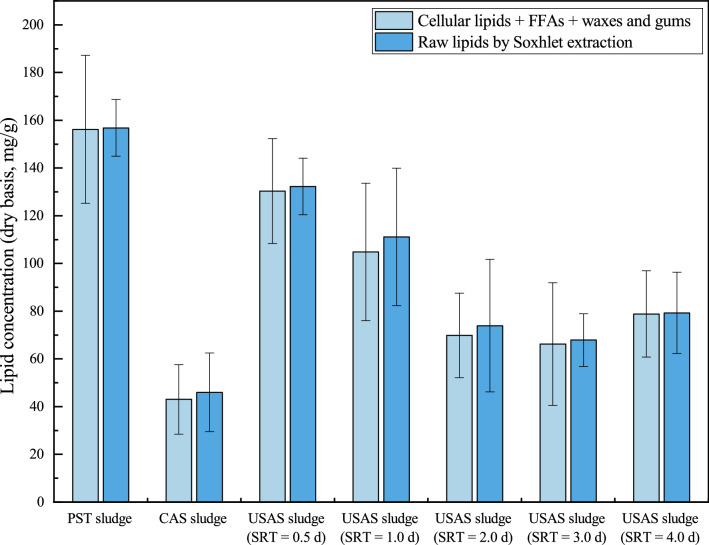
Total lipid concentration of PST sludge, CAS sludge, and USAS sludges.

The total lipid concentrations in different sludge samples are shown in Fig. 1. The total concentration of cellular lipids, FFAs, wax and gum was slightly less than that of raw lipids by Soxhlet extraction, which was consistent with previous studies^19–21^. The lipids contained in the sludge were mainly cellular lipids, FFAs, wax and gum. Compared with the CAS sludge and USAS sludges, the PST sludge contained more lipids. The concentration of raw lipids by Soxhlet extraction of the PST sludge was 156.8 ± 11.9 mg/g, followed by the USAS sludge with the highest concentration (USAS sludge, 0.5 d SRT) of 132.2 ± 11.8 mg/g and an average amount of 92.9 ± 19.3 mg/g, while the concentration for the CAS sludge was 46.0 ± 16.5 mg/g. Particulate organics accounted for more than 50% of the total organics in the influent^22^ and were captured by the primary sedimentation tank^23^. In a typical wastewater treatment plant, the primary sedimentation tank is used to capture suspended organic and inorganic matter, while the biological reaction tank is used to separate colloidal and dissolved organic matter through adsorption and biodegradation from the sewage by activated sludge flocs. Since the CAS sludge was collected from the secondary sedimentation tank of a lab-scale A^2^/O activated sludge system and particulate organics had been captured by the primary sedimentation tank, the organics (mainly dissolved) in the influent were consumed by the microorganisms in the activated sludge, which led to the lower lipid concentration of the CAS sludge. In reports from Frølund et al.^24^ and Wingender et al.^25^, microorganisms accounted for less than 10% of the organic portion of the activated sludge of a conventional activated sludge system, and the remaining portion consisted of extracellular polymeric substances (EPS) secreted by microorganisms. As a result of the high organic loading rate of the USAS system, the microorganisms in the USAS sludge could secrete more EPS than those in the CAS sludge. Considering that the mixed liquid suspended solids (MLSS) of the sludge in the USAS system was less than 1,100 mg/l^26^, which was lower than that of the sludge in the conventional activated sludge system (usually 3,000–5,000 mg/L), under the same influent conditions, it was reasonable that the lipid concentration of the CAS sludge was less than that of the USAS sludge.

### Concentration of three main lipids in the PST, CAS, and USAS sludges

**Figure 2:**
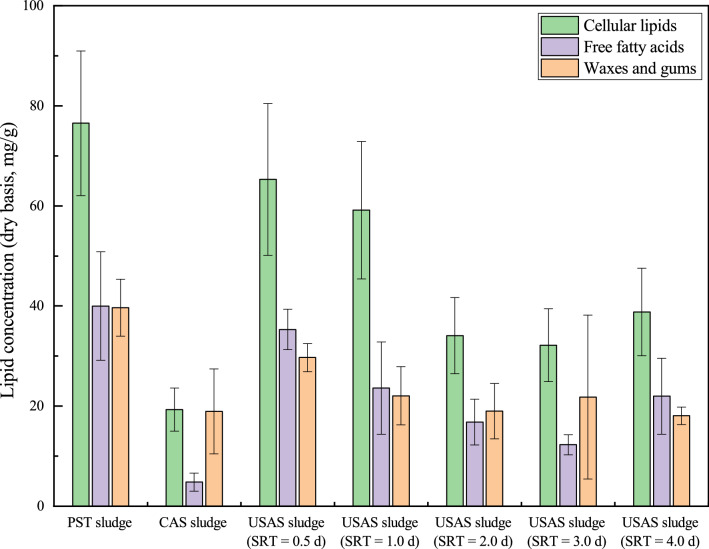
Lipid composition of PST sludge, CAS sludge, and USAS sludges.

It can be observed from Fig. 2 that the compositions of the three main types of lipids in different sludge samples were rather different. In the PST sludge, the concentration of cellular lipids was the highest (76.5 ± 14.4 mg/g), and the concentrations of the other two lipids were almost equal, with 40.0 ± 10.8 mg/g for FFAs and 39.7 ± 5.7 mg/g for wax and gum. In the CAS sludge, the concentrations of the three types of lipids were extremely different. The concentration of FFAs was the lowest (4.8 ± 1.8 mg/g), and the concentration of cellular lipids was highest (19.3 ± 4.3 mg/g), followed by wax and gum (18.9 ± 8.5 mg/g). The concentration of FFAs in PST sludge was much higher than that in CAS sludge, given that FFAs could be a good substrate for assimilation by microorganisms^27^; it can be inferred that most of the FFAs in the sewage were utilized by the microorganisms in the sludge. Considering the capture of wax and gum by the PST, CAS sludge contains less wax and gum. For the sludges from the USAS system, the average amounts of cellular lipids, FFAs, and wax and gum were 45.9 ± 10.5 mg/g, 22.0 ± 5.5 mg/g, and 22.1 ± 6.5 mg/g, respectively. As the lipid compositions of USAS sludges with different SRTs were similar, USAS sludge with a 0.5 d SRT was selected as the typical sludge for further discussion. The lipid composition of the USAS sludges was similar to that of the PST sludge, with cellular lipids having the highest concentration, followed by the other two lipids, which were similar in amount. Research by Meerburg et al.^28^ showed that SRT had a great impact on the community structure and species richness of microorganisms in activated sludge systems. Due to poor system uniformity, the richness of microorganism flora in short-SRT activated sludge systems was lower than that in long-SRT activated sludge systems. According to a report by Gonzalez-Martinez et al.^29^, the core genus of microorganisms in the influent was consistent with those in the USAS systems, while for the CAS sludge, no core genus consistent with the microorganisms in the influent was detected, which indicated that a longer SRT led to a change in microbial community structure in the activated sludge system and thus resulted in a different lipid composition. The lipid composition in the CAS sludge and USAS sludge was found to be rather different, which may be because adsorption played a main role in removing organics from the influent in the USAS system, while the organic matter was mainly removed by microbial degradation in the CAS system.

### Composition of cellular lipids and FFAs in lipids from the PST, CAS, and USAS sludges

**Figure 3:**
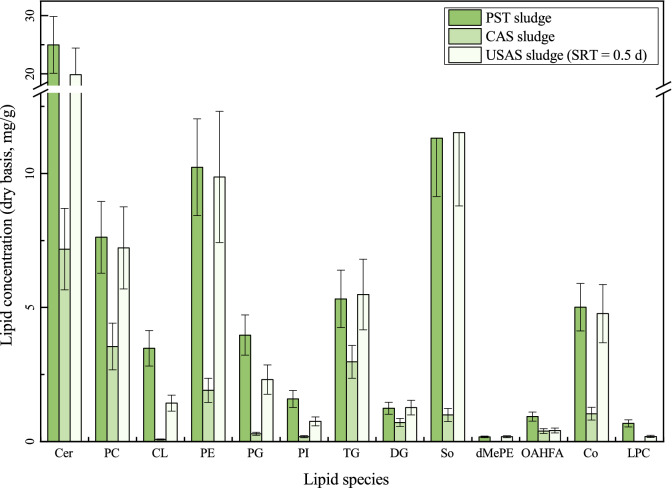
Cellular lipid distribution of PST sludge, CAS sludge, and USAS sludge (0.5 d SRT).

The results of cellular lipid analysis of the PST sludge, CAS sludge, and USAS sludge (0.5 d SRT) are shown in Fig. 3. The concentration of cellular lipids in the other sludges is illustrated in Supplementary Table S1 online. Detection of cellular lipids showed that Cer (25.0 ± 4.9 mg/g), So (11.3 ± 2.2 mg/g), PE (10.2 ± 1.8 mg/g), and PC (7.6 ± 1.3 mg/g) were the most abundant cellular lipids in the PST sludge. For the CAS sludge, the most abundant three cellular lipids were Cer (7.2 ± 1.5 mg/g), PC (3.5 ± 0.9 mg/g), and TG (3.0 ± 0.6 mg/g), while for the USAS sludge (0.5 d SRT), the most abundant three cellular lipids were Cer (19.9 ± 4.6 mg/g), So (11.5 ± 2.7 mg/g), and PE (9.9 ± 2.4 mg/g), followed by PC (7.2 ± 1.5 mg/g). The concentrations of Cer and PC in the three sludges were high, indicating that Cer and PC played a significant role both in the microorganisms of the bioreactor and the organisms of the influent. As elucidated, the sludge from the primary sedimentation tank contained mainly suspended organic and inorganic matter derived from animal and plant residuals in the sewage, while the sludge from the SST was mainly composed of microorganisms and other substances secreted by microorganisms. According to Olkiewicz et al.^30^, phospholipids that make up the cell membrane account for approximately 24–25% of the dry cell mass, and as these components are part of microorganisms, the cellular lipid composition was different due to the different microbial communities. The composition of cellular lipids in the PST sludge and USAS sludge (0.5 d SRT) was similar, especially for phospholipids such as PC, CL, PE, and PG, suggesting that the microbial communities of the two sludges were similar because phospholipids have been reported to identify microbial community in the earth^21^.

**Figure 4:**
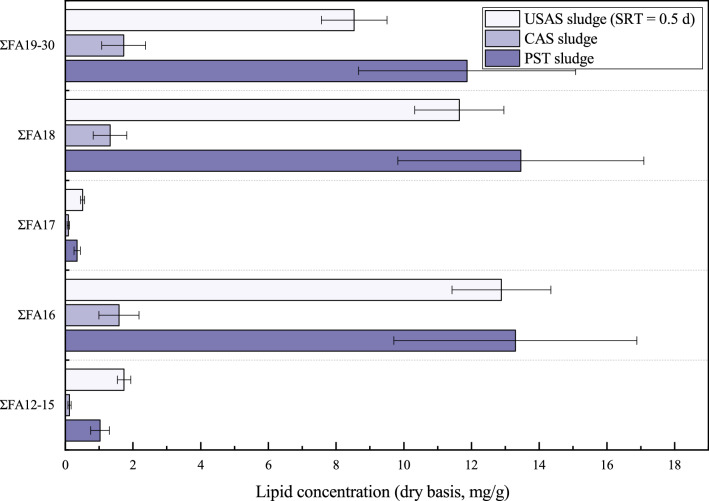
FFA distribution of PST sludge, CAS sludge, and USAS sludge (0.5 d SRT).

Figure 4 shows the FFA distribution in the PST sludge, CAS sludge, and USAS sludge (0.5 d SRT). Information on FA12-FA30 is detailed in Supplementary Table S2 online. Studies^4,6,20^ have shown that the main components of biodiesel made from sludge include palmitic acid (C16: 0), palmitoleic acid (C16: 1), stearic acid (C18: 0), oleic acid (C18: 1), and methyl linoleate (C18: 2), which indicates that C16 or C18 are more suitable as raw materials for biodiesel production. In addition to exhibiting a potential for biodiesel production, sludges containing a considerable amount of FFAs are promising raw materials for fermentation, as they can provide enough volatile fatty acids (VFAs) as a supplementary carbon source in the bioreactor to enhance biological denitrification in WWTPs^31,32^. Considering that FFAs with C16 or C18 are good substrates for producing biodiesel and VFAs, the amount of FA16 and FA18 compounds in sludge can indicate the potential for resource reutilization and energy recovery. The ratio of FA16 + FA18 with respect to total FFAs in the USAS sludge with 0.5 d SRT (69.47%) was even higher than that of the PST sludge (66.89%); therefore, the PST sludge and USAS sludge (0.5 d SRT) have the potential to be used as a source for biodiesel through extraction and purification or through biological enrichment by microorganisms, such as the cultivation of oil-producing microalgae with a substrate of sludge^33^. However, USAS sludge with a 2.0, 3.0, and 4.0 d SRT may not be a good raw material for resource recovery due to the small ratio of FA16 + FA18 or low concentration of FFAs.

### Potential for resource reutilization and energy recovery

**Figure 5:**
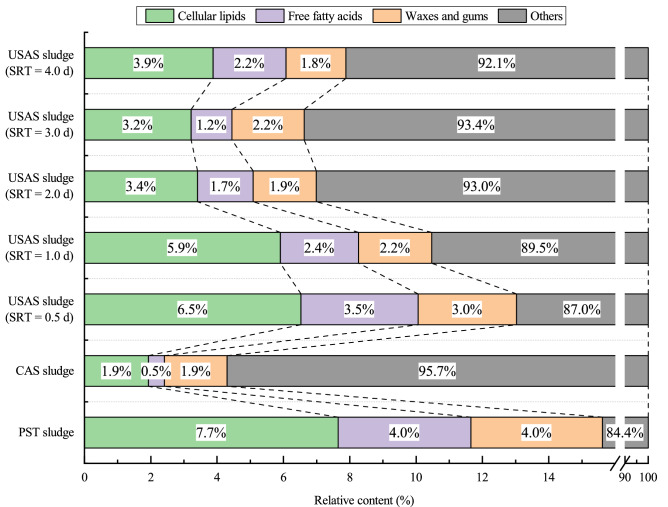
Relative content of lipids in PST sludge, CAS sludge, and USAS sludges.

The content of cellular lipids in the PST sludge (7.65 wt%) was greater than the highest content found in the USAS sludges (6.53 wt%, 0.5 d SRT), and the CAS sludge contained only 1.93 wt% (Fig. 5). The concentrations of Cer, So, PE, and PC in the PST sludge and the USAS sludge (0.5 d SRT) were high; these compounds can be extracted to produce high value-added products, such as cosmetics and biodiesel. For instance, Cer, an important ingredient in cosmetics^34,35^, can be extracted for reutilization, especially from the PST sludge and USAS sludge (0.5 d SRT), which had favorable amounts of Cer (2.50 wt% and 1.99 wt%, respectively, dry basis). Triglycerides (TG), a good raw material for biodiesel production^36–38^, were abundant in the three sludges (PST sludge: 0.53 wt%, CAS sludge: 0.30 wt%, USAS sludge (0.5 d SRT): 0.55 wt%, dry basis); therefore, the sludges had the potential for resource and energy recovery. In addition, other studies^21,39^ have proven the possibility of producing biodiesel with phospholipids as the raw materials; the glyceride phospholipids in sludge include phosphatidylcholine (PC), cardiolipin (CL), phosphatidylethanolamine (PE), and phosphatidylglycerol (PG). The glyceride phospholipid contents in the PST sludge, CAS sludge, and USAS sludge (0.5 d SRT) were 2.53 wt%, 0.58 wt%, and 2.08 wt% (dry basis), respectively, and the glyceride phospholipid contents in the PST sludge and USAS sludge (0.5 d SRT) were high, indicating that the PST sludge and USAS sludge (0.5 d SRT) were promising for producing biodiesel.

**Figure 6:**
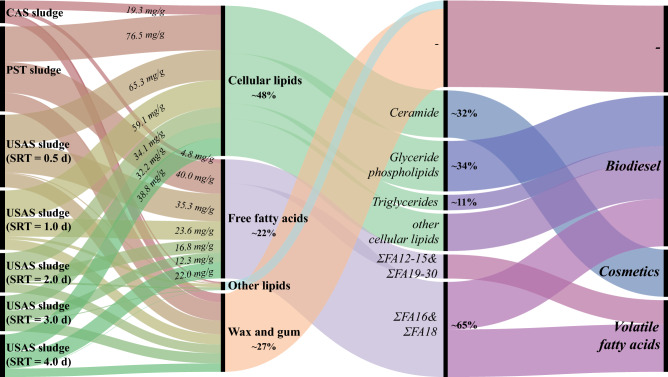
Characteristics and reutilization potential of lipids in the PST, CAS, and USAS sludges.

As shown in Fig. 6, the PST sludge and USAS sludge (0.5 d SRT) had a higher concentration of lipids, especially cellular lipids and FFAs. Ceramide (Cer) and phospholipids (PC + CL + PE + PG) accounted for a large part (~ 32% and ~ 34%, on average) of the cellular lipids, and FA16 + FA18 (~ 65%, on average) was the most abundant species in the FFAs. The possible pathways for resource reutilization and energy recovery were mainly the production of biodiesel and valuable products. Both the glyceride phospholipids and triglycerides in cellular lipids and FA16 + FA18 in FFAs are favorable raw materials for biodiesel production. Cer in sludge can be extracted to manufacture cosmetics. Moreover, sludges with abundant FFAs are suitable for fermentation, providing VFAs as carbon sources for WWTPs.

Economic analysis^7,40^ has shown that with a 10% yield of biodiesel production from sludge, biodiesel will be able to compete with traditional fossil fuel. The yield of biodiesel from municipal WWTP sludge as a raw material is 1.8–6%^41,42^; given that the lipid content of the USAS sludge (9.29%, on average) was approximately twice that of the CAS sludge (4.30%), the production of biodiesel from the USAS sludge would probably be competitive, with a considerable yield higher than 10%. Compared with the other sludges, the PST sludge and USAS sludge (0.5 d SRT) were more suitable for resource reutilization and energy recovery. However, the relative content of lipids in sludge can only represent the efficiency of biodiesel production per unit amount of sludge, given that the MLSS of CAS sludge (usually 3,000–5,000 mg/L) is much larger than that of USAS sludge (less than 1,100 mg/L^26^). Considering that most of the excess sludge in the world is from conventional activated sludge systems and the amount of CAS sludge is extremely large, it can be deduced that CAS sludge can yield a considerable amount of biodiesel.

## Conclusion

This study investigated the lipid characteristics and reutilization potential of sludges from different activated sludge processes. The following conclusions were drawn:

1. Most of the lipids contained in the sludges were cellular lipids, FFAs, wax and gum, accounting for more than 90% of the total lipids. The total concentration of lipids varied among the three types of sludge, and the highest lipid content was found in the PST sludge (156.8 ± 11.9 mg/g, dry basis), followed by the USAS sludges (67.9 ± 11.0–132.2 ± 11.8 mg/g) and the CAS sludge (46.0 ± 16.5 mg/g).
2. In the PST sludge, the content of cellular lipids was the highest (7.65%, dry basis), followed by the contents of FFAs (4.00%) and wax and gum (3.97%). In the USAS sludges, the average contents of cellular lipids, FFAs, and wax and gum were 4.59%, 2.20%, and 2.21%, respectively, while in the CAS sludge, the content of cellular lipids (1.93%) was similar to that of wax and gum (1.89%), and the FFA content was only 0.48%.
3. In these sludge samples, lipid species such as Cer, So, PE, PC, and TG were relatively abundant (over 80%). Among the species of FFAs, FA16 and FA18 accounted for approximately 70% of the total FFAs. The ratio of FA16 + FA18 to total FFAs in the PST sludge, CAS sludge, and USAS sludge (0.5 d SRT) was 66.89%, 59.99%, and 69.47%, respectively.
4. Lipids from these sludges have the potential for resource reutilization and energy recovery. In particular, the PST sludge and USAS sludge (0.5 d SRT) were more favorable raw materials for resource reutilization and energy recovery due to their higher lipid contents (15.68% and 13.22%, dry basis) than the other sludge samples. Moreover, given the large amount of excess sludge, the CAS sludge with a lower lipid content was also promising for resource reutilization and energy recovery.

## Supplementary information

Supplementary information

## References

[1] National Bureau of Statistics (2019). China statistical yearbook on environment-2018 (in Chinese).

[2] Hao, X., Zhang, X., Liu, R. & Hu, R. Bottlenecks and breakthroughs of energy conversion from excess sludge (in Chinese). China Water Wastewater, 4 (2014).

[3] Li Y, Chen T, Luo W, Huang Q, Wu J (2003). Contents of organic matter and major nutrients and the ecological effect related to land application of sewage sludge in China. Acta Ecol. Sin..

[4] Mondala A, Liang K, Toghiani H, Hernandez R, French T (2009). Biodiesel production by in situ transesterification of municipal primary and secondary sludges. Biores. Technol..

[5] Kargbo DM (2010). Biodiesel production from municipal sewage sludges. Energ Fuel.

[6] Revellame E, Hernandez R, French W, Holmes W, Alley E (2010). Biodiesel from activated sludge through in situ transesterification. J. Chem. Technol. Biotechnol..

[7] Revellame E (2011). Production of biodiesel from wet activated sludge. J. Chem. Technol. Biotechnol..

[8] Zhu F (2014). Comparison of the lipid content and biodiesel production from municipal sludge using three extraction methods. Energ Fuel.

[9] Olkiewicz M (2012). Evaluation of different sludges from WWTP as a potential source for biodiesel production. Proc. Eng..

[10] Böhnke, B. Energieminimierung durch das adsorptions-belebungsverfahren. (Gesellschaft zur Förderung der Siedlungswasserwirtschaft an der RWTH, 1981).

[11] Versprille A, Zuurveen B, Stein T (1985). The A-B process: A novel two stage wastewater treatment system. Water Sci. Technol..

[12] Trzcinski AP (2016). Identification of recalcitrant compounds in a pilot-scale AB system: An adsorption (A) stage followed by a biological (B) stage to treat municipal wastewater. Biores. Technol..

[13] Meerburg FA (2015). Toward energy-neutral wastewater treatment: A high-rate contact stabilization process to maximally recover sewage organics. Biores. Technol..

[14] Folch J, Lees M, Sloane SG (1957). A simple method for the isolation and purification of total lipides from animal tissues. J. Biol. Chem..

[15] Bligh EG, Dyer WJ (1959). A rapid method of total lipid extraction and purification. Can. J. Biochem. Physiol..

[16] Rajam L, Soban Kumar D, Sundaresan A, Arumughan C (2005). A novel process for physically refining rice bran oil through simultaneous degumming and dewaxing. J. Am. Oil Chem. Soc..

[17] Huynh L-H, Kasim NS, Ju Y-H (2010). Extraction and analysis of neutral lipids from activated sludge with and without sub-critical water pre-treatment. Biores. Technol..

[18] Tang H (2016). Establishment of local searching methods for orbitrap-based high throughput metabolomics analysis. Talanta.

[19] Jarde E, Mansuy L, Faure P (2005). Organic markers in the lipidic fraction of sewage sludges. Water Res..

[20] Dufreche S (2007). Extraction of lipids from municipal wastewater plant microorganisms for production of biodiesel. J. Am. Oil. Chem. Soc..

[21] Zhu F (2017). Lipid profiling in sewage sludge. Water Res..

[22] Sophonsiri C, Morgenroth E (2004). Chemical composition associated with different particle size fractions in municipal, industrial, and agricultural wastewaters. Chemosphere.

[23] Morgenroth E, Kommedal R, Harremoes P (2002). Processes and modeling of hydrolysis of particulate organic matter in aerobic wastewater treatment—a review. Water Sci. Technol..

[24] Frølund B, Palmgren R, Keiding K, Nielsen PH (1996). Extraction of extracellular polymers from activated sludge using a cation exchange resin. Water Res..

[25] Wingender J, Neu TR, Flemming H-C (1999). Microbial extracellular polymeric substances.

[26] Zhang, Y. Exploratory study on the method of deep separation of organic matters in municipal wastewater (in Chinese) Doctor thesis, Renmin University of China, (2018).

[27] Chipasa KB, Medrzycka K (2008). Characterization of the fate of lipids in activated sludge. J. Environ. Sci..

[28] Meerburg FA (2016). High-rate activated sludge communities have a distinctly different structure compared to low-rate sludge communities, and are less sensitive towards environmental and operational variables. Water Res..

[29] Gonzalez-Martinez, A. et al. Comparison of bacterial communities of conventional and A-stage activated sludge systems. Scientific Reports 6, 18786, doi:10.1038/srep18786. https://www.nature.com/articles/srep18786#supplementary-information (2016). 10.1038/srep18786 PMC4700461 26728449

[30] Olkiewicz M (2014). Direct liquid–liquid extraction of lipid from municipal sewage sludge for biodiesel production. Fuel Process. Technol..

[31] Banister SS, Pretorius W (1998). Optimisation of primary sludge acidogenic fermentation for biological nutrient removal. Water S. A..

[32] Do, P., Amatya, P. L. & Keller, W. E. Successful implementation of biological nutrient removal at Calgary’s 500 ml/d bonnybrook wastewater treatment plant. (2014).

[33] Magdouli S, Yan S, Tyagi RD, Surampalli RY (2014). Heterotrophic microorganisms: A promising source for biodiesel production. Crit. Rev. Environ. Sci. Technol..

[34] Iwai H, Fukasawa J, Suzuki T (1998). A liquid crystal application in skin care cosmetics. Int. J. Cosmet. Sci..

[35] Zhang M, Xie J, Zhou Q, Chen G, Liu Z (2003). On-line solid-phase extraction of ceramides from yeast with ceramide III imprinted monolith. J. Chromatogr. A.

[36] Iso M, Chen B, Eguchi M, Kudo T, Shrestha S (2001). Production of biodiesel fuel from triglycerides and alcohol using immobilized lipase. J. Mol. Catal. B Enzym..

[37] Lestari S, Mäki-Arvela P, Beltramini J, Lu GM, Murzin DY (2009). Transforming triglycerides and fatty acids into biofuels. Chem. Sustain. Energy Mater..

[38] Wu X (2017). Production of jet fuel range biofuels by catalytic transformation of triglycerides based oils. Fuel.

[39] Revellame ED (2012). Parametric study on the production of renewable fuels and chemicals from phospholipid-containing biomass. Top. Catal..

[40] Olkiewicz M, Torres CM, Jimenez L, Font J, Bengoa C (2016). Scale-up and economic analysis of biodiesel production from municipal primary sewage sludge. Biores. Technol..

[41] Revellame ED (2013). Lipid-enhancement of activated sludges obtained from conventional activated sludge and oxidation ditch processes. Biores. Technol..

[42] Wang Y, Feng S, Bai X, Xia S, Peng D (2015). Research status of biodiesel production from wastewater sludge by methanolysis (in Chinese). China Water Wastewater.

