# Supplementary material for: Characterization and reutilization potential of lipids in sludges from wastewater treatment processes
Source: Sci Rep. 2020 Aug 3;10:12997. doi: 10.1038/s41598-020-69855-6 (PMC7400647; doi:10.1038/s41598-020-69855-6)
Supplement: Supplementary file 1 — Supplementary information [file 41598_2020_69855_MOESM1_ESM.docx]

**Characterization and reutilization potential of lipids in sludges from wastewater treatment processes**

**Shuai Liu**^#^**, Tao Luo**^#^**, Guo-hua Liu*****, Xianglong Xu, Yuting Shao, Lu Qi, Hongchen Wang****

Low-carbon Water Environment Technology Research Center, School of Environment & Natural Resources, Renmin University of China, Beijing, 100872, China

E-mail:

Shuai Liu: 2015200697@ruc.edu.cn

Tao Luo: luotao526@ruc.edu.cn

^#^These two authors are co-first authors

*Corresponding authors: Guo-hua Liu and Hongchen Wang

E-mail: lgh@ruc.edu.cn and whc@ruc.edu.cn

Tel: +86-10-62510853

Fax: +86-10-62510853

**Supplementary information I**

**Table S1 Cellular lipids content in the sludge**

| Lipid concentration (mg/g) | PST sludge | CAS sludge | USAS sludge | | | | |
| --- | --- | --- | --- | --- | --- | --- | --- |
| SRT | - | 12 d | 0.5 d | 1 d | 2 d | 3 d | 4 d |
| Cer | 24.96±4.89 | 7.18±1.52 | 19.86±4.57 | 18.31±4.11 | 10.38±2.22 | 8.91±1.99 | 12.33±3.04 |
| PC | 7.62±1.34 | 3.54±0.87 | 7.23±1.53 | 6.33±1.65 | 8.95±2.06 | 4.54±1.00 | 6.27±1.42 |
| CL | 3.48±0.66 | 0.08±0.02 | 1.44±0.3.0 | 1.83±0.47 | 0.08±0.02 | 0.91±0.21 | 0.17±0.04 |
| PE | 10.23±1.81 | 1.91±0.45 | 9.87±2.45 | 9.42±2.03 | 3.76±0.80 | 3.70±0.91 | 6.34±1.42 |
| PG | 3.97±0.75 | 0.29±0.06 | 2.31±0.54 | 2.83±0.75 | 0.24±0.05 | 1.44±0.34 | 0.33±0.07 |
| PI | 1.59±0.32 | 0.18±0.04 | 0.75±0.17 | 1.18±0.29 | 0.15±0.04 | 0.85±0.18 | 0.19±0.04 |
| TG | 5.32±1.07 | 2.97±0.61 | 5.48±1.31 | 5.54±1.42 | 3.94±0.92 | 4.26±1.04 | 5.91±1.20 |
| DG | 1.24±0.22 | 0.71±0.15 | 1.27±0.27 | 1.22±0.27 | 2.01±0.44 | 1.01±0.25 | 1.22±0.28 |
| So | 11.32±2.17 | 0.99±0.24 | 11.52±2.74 | 10.6±2.28 | 2.83±0.70 | 5.39±1.13 | 5.67±1.14 |
| dMePE | 0.17±0.03 | 0.01±0.00 | 0.18±0.04 | 0.12±0.03 | 0.02±0.01 | 0.04±0.01 | 0.02±0.00 |
| OAHFA | 0.93±0.17 | 0.39±0.09 | 0.42±0.09 | 0.29±0.07 | 0.23±0.06 | 0.11±0.02 | 0.03±0.01 |
| Co | 5.01±0.89 | 1.04±0.23 | 4.77±1.09 | 1.39±0.37 | 1.49±0.31 | 0.66±0.15 | 0.28±0.06 |
| LPC | 0.68±0.13 | 0.13±0.01 | 0.01±0.00 | 0.00±0.19 | 0.19±0.04 | 0.04±0.10 | 0.10±0.02 |
| Total cellular lipids | 76.52±14.45 | 19.3±4.30 | 65.29±15.14 | 59.15±13.74 | 34.08±7.61 | 32.17±7.28 | 38.78±8.73 |

**Supplementary information** **Ⅱ**

**Table S2 Free fatty acid (FA12-FA30) content in the sludge**

| Lipid content | PST sludge | CAS sludge | USAS sludge | | | | |
| --- | --- | --- | --- | --- | --- | --- | --- |
| SRT | - | 12 d | 0.5 d | 1 d | 2 d | 3 d | 4 d |
| **Absolute content(mg/g)** | | | | | | | |
| ΣFA | 40.0 ± 10.8 | 4.8 ± 1.8 | 35.3 ± 4.0 | 23.6 ± 9.2 | 16.8 ± 4.6 | 12.3 ± 2.0 | 22.0 ± 7.6 |
| **Relative content(%)** | | | | | | | |
| FA(12:0) | 0.14 | 0.08 | 0.16 | 0.16 | 0.09 | 0.03 | 0.08 |
| FA(13:0) | 0.02 | 0.02 | 0.11 | 0.18 | 0.06 | 0.03 | 0.03 |
| FA(14:0) | 1.96 | 1.89 | 1.70 | 1.47 | 0.96 | 0.64 | 1.17 |
| FA(14:1) | 0.05 | 0.04 | 0.23 | 0.26 | 0.20 | 0.13 | 0.10 |
| FA(15:0) | 0.38 | 0.54 | 2.26 | 3.19 | 1.64 | 1.50 | 1.13 |
| FA(15:1) | 0.02 | 0.02 | 0.46 | 0.47 | 0.36 | 0.38 | 0.16 |
| FA(16:0) | 32.36 | 25.57 | 25.05 | 22.39 | 22.15 | 25.67 | 23.73 |
| FA(16:1) | 0.54 | 4.20 | 10.08 | 13.61 | 13.39 | 18.09 | 10.03 |
| FA(16:1)*1 | 0.31 | 3.21 | 0.48 | 0.57 | 0.30 | 0.02 | 0.28 |
| FA(16:2) | 0.04 | 0.04 | 0.86 | 0.91 | 0.52 | 0.71 | 0.29 |
| FA(16:3) | 0.00 | 0.00 | 0.02 | 0.04 | 0.04 | 0.04 | 0.03 |
| **ΣFA16** | **33.24** | **33.03** | **36.50** | **37.51** | **36.40** | **44.52** | **34.36** |
| **饱和度** | **97.33** | **77.42** | **68.63** | **59.68** | **60.86** | **57.63** | **69.05** |
| FA(17:0) | 0.71 | 1.64 | 0.72 | 0.82 | 0.55 | 0.37 | 0.41 |
| FA(17:1) | 0.11 | 0.13 | 0.62 | 0.92 | 0.47 | 0.12 | 0.30 |
| FA(17:1)*1 | 0.05 | 0.13 | 0.11 | 0.11 | 0.07 | 0.04 | 0.08 |
| FA(18:0) | 19.2 | 13.72 | 16.01 | 13.78 | 14.23 | 10.19 | 13.59 |
| FA(18:1) | 6.63 | 4.99 | 12.21 | 14.35 | 12.06 | 11.06 | 7.26 |
| FA(18:1)*1 | 5.94 | 4.71 | 3.38 | 3.85 | 1.92 | 0.72 | 1.17 |
| FA(18:2) | 1.88 | 3.15 | 1.34 | 0.98 | 1.63 | 1.40 | 1.06 |
| FA(18:4) | 0.00 | 0.00 | 0.03 | 0.05 | 0.04 | 0.08 | 0.02 |
| **ΣFA18** | **33.65** | **27.57** | **32.97** | **33.00** | **29.87** | **23.44** | **23.11** |
| **饱和度** | **57.07** | **47.91** | **48.56** | **41.75** | **47.63** | **43.48** | **58.80** |
| **ΣFA16+ΣFA18** | **66.89** | **60.60** | **69.48** | **70.51** | **66.27** | **67.96** | **57.47** |
| **总饱和度** | **77.08** | **63.99** | **59.10** | **51.29** | **54.90** | **52.74** | **64.93** |
| FA(19:0) | 0.14 | 0.14 | 0.13 | 0.12 | 0.09 | 0.06 | 0.07 |
| FA(19:1) | 0.06 | 0.10 | 0.25 | 0.36 | 0.19 | 0.24 | 0.18 |
| FA(19:1)*1 | 0.05 | 0.07 | 0.14 | 0.20 | 0.10 | 0.12 | 0.07 |
| FA(20:0) | 3.62 | 3.49 | 2.68 | 2.24 | 2.52 | 1.54 | 2.68 |
| FA(20:1) | 0.38 | 0.37 | 0.40 | 0.43 | 0.32 | 0.21 | 0.23 |
| FA(20:1)*1 | 0.26 | 0.29 | 0.20 | 0.18 | 0.17 | 0.11 | 0.11 |
| FA(20:2) | 0.07 | 0.06 | 0.08 | 0.07 | 0.07 | 0.04 | 0.03 |
| FA(20:3) | 0.01 | 0.02 | 0.04 | 0.09 | 0.04 | 0.06 | 0.02 |
| FA(20:4) | 0.05 | 0.04 | 0.20 | 0.27 | 0.40 | 0.52 | 0.12 |
| FA(20:5) | 0.01 | 0.01 | 0.23 | 0.17 | 0.79 | 3.64 | 0.55 |
| FA(21:0) | 0.72 | 0.75 | 0.59 | 0.55 | 0.56 | 0.43 | 0.60 |
| FA(22:0) | 9.28 | 10.72 | 7.26 | 6.10 | 8.21 | 6.51 | 10.57 |
| FA(22:1) | 0.41 | 0.42 | 0.30 | 0.28 | 0.33 | 0.27 | 0.29 |
| FA(22:2) | 0.01 | 0.03 | 0.03 | 0.03 | 0.02 | 0.01 | 0.01 |
| FA(22:4) | 0.01 | 0.01 | 0.03 | 0.04 | 0.03 | 0.06 | 0.01 |
| FA(22:5) | 0.01 | 0.01 | 0.05 | 0.06 | 0.12 | 0.41 | 0.06 |
| FA(22:6) | 0.02 | 0.02 | 0.13 | 0.11 | 0.21 | 0.51 | 0.16 |
| FA(23:0) | 2.18 | 2.54 | 1.66 | 1.44 | 1.85 | 1.49 | 2.47 |
| FA(23:1) | 0.12 | 0.20 | 0.11 | 0.12 | 0.11 | 0.09 | 0.12 |
| FA(24:0) | 5.89 | 7.27 | 4.31 | 4.28 | 6.12 | 5.58 | 9.63 |
| FA(24:1) | 0.79 | 0.79 | 0.61 | 0.56 | 0.64 | 0.59 | 0.67 |
| FA(24:2) | 0.09 | 0.09 | 0.07 | 0.06 | 0.07 | 0.05 | 0.05 |
| FA(24:5) | 0.07 | 0.19 | 0.04 | 0.02 | 0.02 | 0.01 | 0.05 |
| FA(24:6) | 0.04 | 0.13 | 0.03 | 0.01 | 0.01 | 0.01 | 0.03 |
| FA(25:0) | 1.23 | 1.74 | 0.90 | 0.82 | 1.29 | 1.12 | 1.91 |
| FA(25:1) | 0.10 | 0.12 | 0.08 | 0.07 | 0.08 | 0.08 | 0.10 |
| FA(25:2) | 0.02 | 0.04 | 0.02 | 0.02 | 0.02 | 0.01 | 0.03 |
| FA(26:0) | 1.92 | 2.71 | 1.65 | 1.51 | 2.31 | 2.20 | 3.74 |
| FA(26:1) | 0.22 | 0.26 | 0.17 | 0.16 | 0.21 | 0.21 | 0.30 |
| FA(26:2) | 0.12 | 0.17 | 0.10 | 0.07 | 0.09 | 0.07 | 0.13 |
| FA(26:3) | 0.04 | 0.08 | 0.04 | 0.03 | 0.04 | 0.03 | 0.07 |
| FA(26:4) | 0.00 | 0.01 | 0.02 | 0.03 | 0.08 | 0.53 | 0.02 |
| FA(27:0) | 0.26 | 0.38 | 0.24 | 0.22 | 0.33 | 0.31 | 0.56 |
| FA(27:1) | 0.03 | 0.04 | 0.02 | 0.02 | 0.03 | 0.03 | 0.04 |
| FA(27:3) | 0.03 | 0.04 | 0.02 | 0.02 | 0.03 | 0.05 | 0.03 |
| FA(27:5) | 0.04 | 0.04 | 0.04 | 0.03 | 0.02 | 0.02 | 0.02 |
| FA(28:0) | 0.63 | 1.24 | 0.64 | 0.54 | 0.90 | 0.75 | 1.69 |
| FA(28:1) | 0.12 | 0.14 | 0.09 | 0.09 | 0.13 | 0.13 | 0.18 |
| FA(29:0) | 0.09 | 0.14 | 0.08 | 0.07 | 0.10 | 0.10 | 0.20 |
| FA(30:0) | 0.31 | 0.52 | 0.28 | 0.24 | 0.41 | 0.36 | 0.81 |
| FA(30:1) | 0.13 | 0.17 | 0.12 | 0.11 | 0.17 | 0.17 | 0.22 |
| FA(30:3) | 0.06 | 0.21 | 0.06 | 0.03 | 0.07 | 0.04 | 0.19 |
| FA(30:4) | 0.02 | 0.07 | 0.02 | 0.01 | 0.02 | 0.02 | 0.06 |
| FA(30:5) | 0.01 | 0.04 | 0.02 | 0.01 | 0.01 | 0.01 | 0.03 |

**Supplementary information Ⅲ**

**Table S3 Gradient elution program**

| Time/min | A% | B% |
| --- | --- | --- |
| 0 | 68 | 32 |
| 1.5 | 68 | 32 |
| 5 | 48 | 52 |
| 18 | 25 | 75 |
| 21 | 3 | 97 |
| 25 | 3 | 97 |
| 25.1 | 68 | 32 |
| 30 | 68 | 32 |
